# Study on the Abrasive Blasting Mechanism of Solder Welded 304V Wire in Vascular Intervention

**DOI:** 10.3390/mi15121405

**Published:** 2024-11-21

**Authors:** Yao Liu, Shaobo Zhai, Jinzhu Guo, Shiling Fu, Bin Shen, Zhigang Zhao, Qingwei Ding

**Affiliations:** 1 Shanxi Key Lab of Advanced Manufacturing Technology, North University of China, Taiyuan 030051, China; liuyao@nuc.edu.cn (Y.L.); z13834038164@163.com (S.Z.); 18234146352@163.com (J.G.); zhao105820@163.com (Z.Z.); 2 Guangdong Provincial Key Laboratory of Minimally Invasive Surgical Instruments and Manufacturing Technology, Guangdong University of Technology, Guangzhou 510006, China; 3 Jiaxing Jiangxin Medical Technology Co., Ltd., Jiaxing 314200, China; fu18823128633@hotmail.com (S.F.); binshen@zgjczg.com (B.S.); 4 Yangtze Delta Region Institute of Tsinghua University, Jiaxing 314000, China; 5 Department of Vascular Surgery, General Surgery Clinical Center, Shanghai General Hospital, Shanghai Jiao Tong University School of Medicine, 100 Haining Road, Shanghai 200080, China

**Keywords:** abrasive blasting, orthogonal experiment, surface roughness, interventional devices

## Abstract

The solder burrs on the 304V wire surface can easily scratch the vascular tissue during interventional treatment, resulting in complications such as medial tears, bleeding, dissection, and rupture. Abrasive blasting is often used to remove solder burr and obtain a smooth surface for the interventional device. This study conducted an abrasive blasting experiment to explore the effects of process parameters (air pressure, lift-off height, abrasive volume, and abrasive type) on processing time, surface roughness, and mechanical properties to reveal the material removal mechanism. The results indicated that the resin abrasive can remove the SAC burr and keep the 304V integrity due to the proper hardness and Young’s module. Impaction pits are the main material removal mode in abrasive blasting. The processing time decreases with the increase in air pressure. The surface roughness increases with the increase in abrasive volume. The primary and secondary factors affecting the surface roughness of the 304V wire after abrasive blasting are the abrasive type and air pressure, followed by the abrasive volume and lift-off height. Blasting leads to a decrease in yield strength, and Young’s modulus and the hardness of the abrasive will affect the tensile strength. This study lays a foundation for understanding abrasive blasting and different cutting mechanisms.

## 1. Introduction

Guidewire [1,2], stent [3], filter [4], interventional coil [5], and braided catheter, used in vascular intervention, are made of precision wire. The connection between the wire and other components, such as steel tube, endoscopic lens house, radiopaque marker bands, and coil, is usually soldered. Due to the natural solidification of the solder after melting and the effect of gravity, burrs is easy to generate. In the vascular intervention, the burrs may scratch the intima and media of the blood vessels, resulting in hemorrhagic thrombosis, intima endometrial, media tearing, and dissection. In severe cases, it may cause vascular perforation and fracture, which may cause myocardial infarction and death. It is necessary to remove the residual solder burr to generate a smooth surface. The grinding and abrasive blasting are the two main ways to deburr the solder. Grinding is usually used for a regular shape with uniform dimensions. However, for heterogeneous structures, large dimensional variation, and sharp corner surfaces such as vascular filters, stents, and ventricular occlude, burrs on the corners cannot be reached by the wheel and can only be removed by abrasive blasting.

The abrasive blasting process uses compressed air to drive an abrasive to impact and scratch the workpiece surface at high speed [6]. Many factors affect the performance of the workpiece after abrasive blasting, including the size and shape of the abrasive particles, the jet pressure, the lift-off height, the impact velocity, and so on. The parameters for evaluating the performance of the workpiece include surface roughness, surface properties, residual stress, mechanical properties, etc. The existing literature focuses on the relationship between them.

Some scholars have conducted in-depth research on the specific influence mechanisms of the individual factors. Li et al. [7] found that abrasive blasting destroys the surface integrity of single-crystal superalloys, resulting in irregular pits on the surface caused by the cutting by abrasive particles while also changing the surface morphology; the surface roughness and microhardness increase as sand particle size increases. Masanoa et al. [8] evaluated the bending strength of four zirconia materials after abrasive blasting using the Al2O3 abrasive and found that microcracks appeared on the subsurface with high compressive stress. Cruz et al. [9] pointed out that pressure is the key factor in workpiece modification; it accounts for 57.0%, 72.8%, and 61.0% of the change in roughness, hardness, and corrosion resistance, respectively. Ding et al. [10] found that the surface layer of titanium after 200–300 μm SiO_2_ abrasive blasting was transformed into a nanocrystalline structure, which improved corrosion resistance and fatigue strength. Based on Abaqus, Cao et al. [11] pointed out that the higher the shot peening intensity, the greater the depth corresponding to the residual stress field of the target, and the more serious the surface roughness. Some scholars have analyzed the effect of two different factors on the performance of the workpiece. Anna [12] pointed out that the abrasive type has a larger influence on the surface roughness and adhesion of the workpiece than the air pressure in abrasive blasting. Lundgren et al. [13] evaluated the tooth enamel damage degree caused by abrasive blasting and pointed out that time a has stronger influence than that of lift-off height. Some scholars have carried out multi-factor analysis by carrying out experiments and constructing related models. Laureniu et al. [14] constructed a mathematical model to predict the abrasive blasting surface roughness, which is affected by lift-off height, abrasive size, and impact angle. Kanesan et al. [15] studied the erosion characteristics of abrasive blasting on 316 stainless steel wire screen meshes. The wear of the workpiece becomes more serious with the increase in the abrasive particle size and impact speed, and there was a shift in the erosion mechanism from micro-plowing to deeper plowing and in the pitting mechanism when the impact angle increased from 30° to 45°. However, the magnitude of plowing and pitting reduced as the impact angle increased to 60° and 90°. Budi et al. [16] studied the influence mechanism of three different abrasives (metallic shot, slag balls, and silica particles) on the surface morphology of 316L stainless steel in abrasive blasting, and the surface roughness value caused by the silica particles that were angular and had an irregular surface morphology was the largest. Guo et al. [17] used six different abrasives with different sizes to blast titanium plates, and they exhibited different characteristics. Carlos et al. [18] pointed out that the residual stress generated during ceramic abrasive blasting is the main factor that causes surface defects.

In the removal of solder burr by abrasive blasting, the abrasive also removes the substrate material, which causes a decrease in the strength and fatigue life. Therefore, the process of different cutting, which is to remove the solder and keep the integrity of the substrate as much as possible, should be investigated via a systematic analysis of the interaction between multiple factors of sandblasting, which has not been reported in the studies reviewed above. Orthogonal tests are usually used for multi-factor analysis, which is another design method to study multiple factors and multiple levels. It selects some representative points from the comprehensive test according to the orthogonality and can reduce the test time and cost, while ensuring the reliability and repeatability of the experimental results.

In this study, abrasive blasting experiments were conducted to reveal the differential cutting mechanism of 304V wire and SAC305. Firstly, the abrasive blasting experimental setup was introduced, and Taguchi experiments were conducted to investigate the effect of air pressure, lift-off height, abrasive volume, and the abrasive (including type and size). Then, the surface topography was observed to reveal the removal mechanism in abrasive blasting. The processing time for the SAC removal, the surface roughness, and the tensile and yield strength of the wire component after abrasive blasting were measured to reveal the removal mechanism indirectly. Finally, the conclusions were drawn regarding the significance of this study.

## 2. Experimental Setup

The wire component is a spring shaft (outer diameter: 0.65 mm) coiled by a 304V stainless steel wire (diameter: 0.15 mm), which is the most used metal for medical devices. The solder is SAC305, which is 96.5% tin, 3% silver, and 0.5% copper in weight, and has been proven to have no toxicity for the human body. The melted SAC305 adheres to the wire component surface and further realizes the connection to other components. Table 1 presents the size and mechanical properties of the wire component and solder.

**Figure 1:**
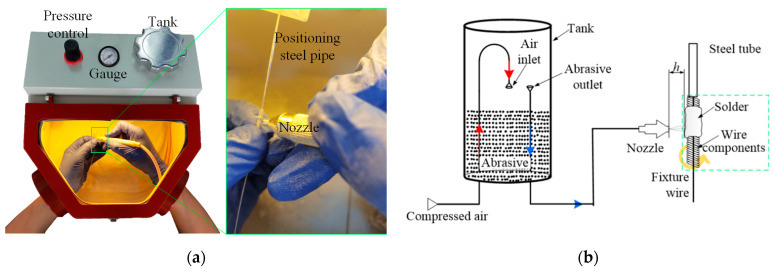
Abrasive blasting experimental setup. (**a**) Overview and (**b**) configuration.

The device used in this experiment is a single-pen abrasive blasting machine (BP-1, Tianjin Lizhong Huier Technology Development Co., Ltd., Tianjin, China), which uses compressed air to carry the abrasive in dry conditions. The abrasive tank is a cylindrical container with a diameter of 70 mm and a height of 170 mm. There is an abrasive delivery pipeline with a height of 110 mm in the tank. The height of the abrasive should not exceed the exit tube of the container. The compressed air from the pipeline inlet impacts on and mixes with the abrasives. The mixture was taken from the outlet to the nozzle. The abrasive in the mixture flow with the air. Then, the abrasive impacts the workpiece, punching and scratching it, as shown in Figure 1.

During the experiment, to figure out the effect of compressed air, the lift-off height of the nozzle, and the abrasive volume and type on the removal process, the Taguchi experiments were designed and conducted. The resin abrasive used was thermosetting urea-formaldehyde (UF) resin [19]. The plastic abrasives used were thermoplastic polyurethane rubber (TPU), polyvinyl chloride (PVC), and polytetrafluoroethylene (PTFE). The ground corn kernels and walnut shells were used as representations of the biomass material. An industry alumina abrasive with high hardness was chosen as the last one. Based on the number and level of the factors, the standard L27 (3^9^ × 9^1^) experiment table was designed, as shown in Table 2.

The range analyses are often used to assess the effect of the factors. The larger the range, the greater the effect of the factors. The calculation of the range *R* is as follows:

(1) $$R={\bar{K}}_{\max}-{\bar{K}}_{\min},$$

where ${\bar{K}}_{\max}$ and ${\bar{K}}_{\min}$ are the maximum and the minimum meaning of results at specified factors with different levels. The $\bar{K}$ can be calculated as follows:

(2) $$\bar{K}=\frac{K}{i},$$

where *K* is the sum of the experimental results of factor level, and *i* is the number of factor levels in the experiment; *i* = 3 or 9 for this study.

In this study, the air pressures were selected as 0.3, 0.4, and 0.5 MPa. The lift-off heights were 2, 4, and 6 mm, which is controlled by a rule before the blasting. The abrasive volumes in height were 60, 80, and 100 mm. A total of 9 types of abrasive were selected in this experiment, as shown in Table 1, which were easily obtained from the market. To achieve the uniform removal of the solder, the 304V wire component was rotated at a constant speed of 80°/s during abrasive blasting. A magnifying glass was set on the 304V wire to check the remaining solder, and the minimum time to remove all solder was recorded with a stopwatch. To reduce the potential damage to the wire component, the maximum abrasive blasting time is set at 300 s. When the abrasive size or type was changed, the tank and pipeline were washed with water and dried with compressed air.

**Figure 2:**
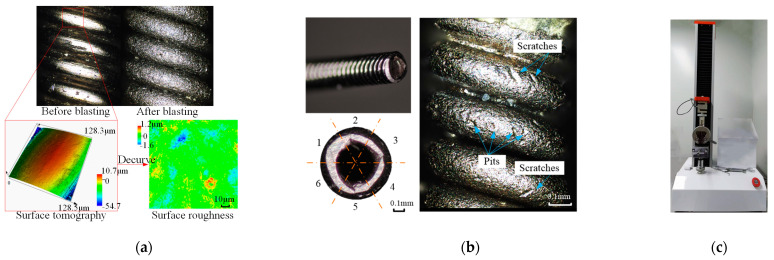
Measurement of test results: (**a**) surface roughness, (**b**) surface damage, and (**c**) mechanical properties.

A laser confocal microscope LEXT OLS4000 (Olympus, Tokyo, Japan) was used to observe the surface after abrasive blasting. The solder’s removal condition was double-checked. The experiment samples that could not remove the solder completely are excluded in the following discussion. The surface roughness of the wire was measured by a laser confocal microscope LEXT OLS4000 (Olympus, Tokyo, Japan) before and after the abrasive blasting. Polynomial filtering was used to remove the wire curvature for surface roughness measurement. Five points on each sample were measured, and the average value was calculated. The original surface roughness of the 304 V wire before the abrasive blasting was 0.16 μm, as shown in Figure 2a. Six photos uniformly distributed on the wire surface were taken for damage checking, as shown in Figure 2b. The pits with a diameter larger than 10 μm and the scratches with a length greater than 50 μm on the surface were counted to evaluate the wire damage after abrasive blasting, as shown in Figure 2b. The mechanical properties of the wire components after abrasive blasting were measured with a tensile test. As shown in Figure 2c, the initial length of the wire was 100 mm, and the feed rate was 200 mm/min. Ten tensile tests were conducted on the wire component after abrasive blasting.

## 3. Results

### 3.1. Surface Topography

**Figure 3:**
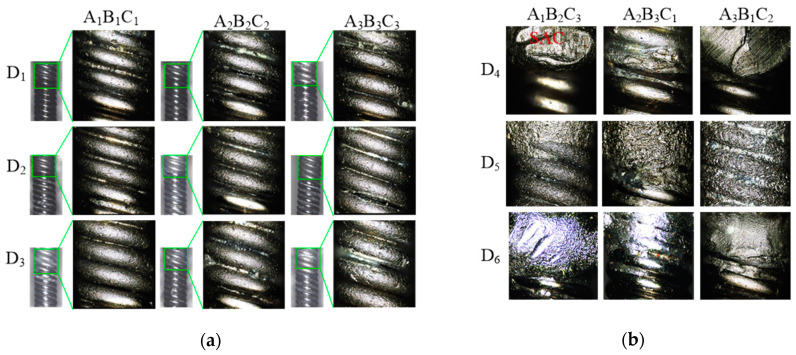
Blasting surface topography by (**a**) resin and (**b**) plastic abrasive.

**Figure 4:**
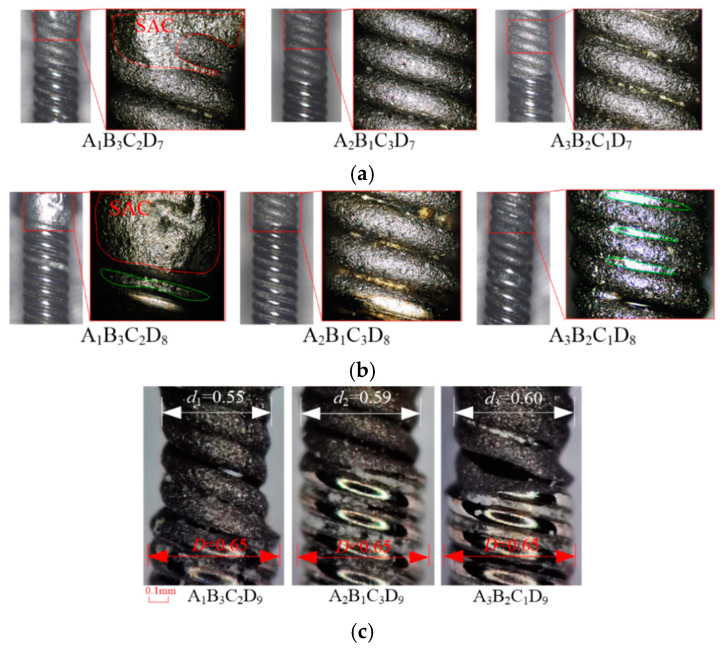
Blasting surface topography by (**a**) corn, (**b**) walnut, and (**c**) alumina abrasive.

Figure 3 and Figure 4 show the 304V wire components after abrasive blasting. The resin abrasive in nine tests can effectively remove all the SAC 305 solder on the surface, as shown in Figure 3a. The size of the wire is the same before and after abrasive blasting, which shows the poor removal ability of the resin abrasive on the 304V wire. The smooth wire surface after abrasive blasting shows many pits, which cause the loss of metallic luster. The plastic abrasives in nine tests fail to remove all the solder on the wire surface, as shown in Figure 3b. The solder after abrasive blasting by a plastic abrasive was not subjected to any obvious impact, which indicates a poor removal ability. The PVC (D5) abrasive showed a stronger ability to remove solder than the TPU (D4) and PTFE (D6) abrasives after 300 s, which also partially changed the wire surface topography obviously.

The biomass abrasive (corn (D7) and walnut (D8)) cannot remove the SAC 305 on the wire surface completely, as shown in Figure 4a,b. Due to the poor material removal ability, all the abrasive blasting times are 300 s. Under the same processing time, the remaining SAC305 area on the wire surface after processing by corn is smaller than that of walnut. The wire diameter after processing by the resin, plastic, and biomass abrasives is not changed, except for the many impact pits. The impact from the abrasive can generate compressive stress on the wire surface, which can improve the fatigue life of the wire theoretically. The SAC305 was partially or completely removed for those kinds of abrasive, even if the efficiency may not have been satisfactory. However, the wire diameter did not change, which showed the different cuttings. A reduction in the diameter of the wire components was observed after abrasive blasting by the alumina (D9) abrasive, after the SAC305 was removed. As shown in Figure 4c, the diameters of the 304V components after abrasive blasting were 0.55 mm, 0.59 mm, and 0.60 mm, which is smaller than the original 0.65 mm. This diameter reduction will weaken the wire strength and result in a potential fracture in future applications.

### 3.2. Processing Time

Table 2 shows the processing time in each test. The TPU (D4), PVC (D5), and PTFE (D6) plastic abrasives did not remove all the SAC305 on the wire surface within 300 s, indicating a weak removal ability. The average processing time of the alumina abrasive (D9) was 6.67 s, which is much less than that of the other eight abrasives, indicating a strong material removal ability. However, the short processing time of the alumina abrasive was detrimental to the control of the removal process, inducing great damage to the wire components. The processing times of the resin abrasives (D1, D2, D3) under different mesh sizes and process parameters were in the range of 35 to 170 s. The time distribution range is 135 s. The large time range makes adjustment easy. The processing time decreases with the decrease in abrasive size. In the six tests (No. 7, 8, 11, 12, 22, and 24) using corn (D7) and walnut (D8) abrasives, the SAC305 can be removed, except in No. 7 and 8, which failed to remove all SAC305 in 300 s. The processing time was between 180 and 300 s. This shows that the removal material ability of the corn and walnut abrasives was greatly affected by the process parameters. The overall processing time was long, resulting in low production efficiency.

**Figure 5:**
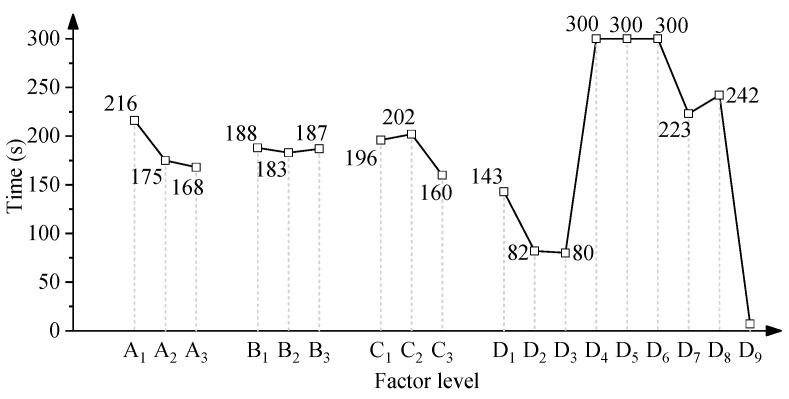
Effect of factor level on processing time.

The factor analysis by using the processing time is calculated and shown in Figure 5. The processing time decreases with the increase in air pressure. The high air pressure results in high abrasive velocity and impact energy. The lift-off height shows no effect on the processing time. The abrasive volume under 80 mm shows no difference in the processing time. However, when the abrasive volume increases to 100 mm, the processing time decreases by 20% to 160 s. A higher abrasive volume can obtain a shorter processing time, due to an increase in the impact frequency, which can remove solder more efficiently. The abrasive types have a great effect on the processing time. The resin can remove the solder in an acceptable range. However, the plastic cannot remove the solder within 300 s. The biomass abrasive can remove the solder after 200 s, which shows lower efficiency than the resin abrasive. The alumina abrasive can eliminate the solder very quickly, which makes it hard to control the time and result in the wire removal.

### 3.3. Surface Roughness

After the SAC305 is removed, the 304V wire surface is exposed and directly impacted by the abrasive, which leads to the deformation and change in surface roughness. The surface roughness of the wire after sandblasting is shown in Table 2. The roughness of testing No. 5, 7, 8, 16, 17, 19, 20, and 21, which can only partially remove SAC305 solder within 300 s, is measured on the exposure area. Tests No. 4, 6, and 18 have an insufficient ability to remove soldering, and the wire is still buried in the SAC305, due to the poor removal ability in the limited time. Therefore, the surface roughness adopts the initial roughness. The resin abrasive in the nine tests shows the smallest surface roughness. The maximum 304V surface roughness after sandblasting is 0.62 μm, indicating little damage to the wire substrate. The use of the resin abrasive can effectively remove solder without damaging the 304V wire, which is more suitable for differential cutting. The surface roughness caused by the alumina abrasive ranges from 0.44 to 0.83 µm, which is higher than that of the resin abrasive. The corn and walnut abrasives are ground from biomass, resulting in an irregular shape, which in turn leads to higher surface roughness. The surface roughness of tests No. 5 and 19, which use the PVC abrasive, is 1.02 μm and 0.74 μm, respectively.

**Figure 6:**
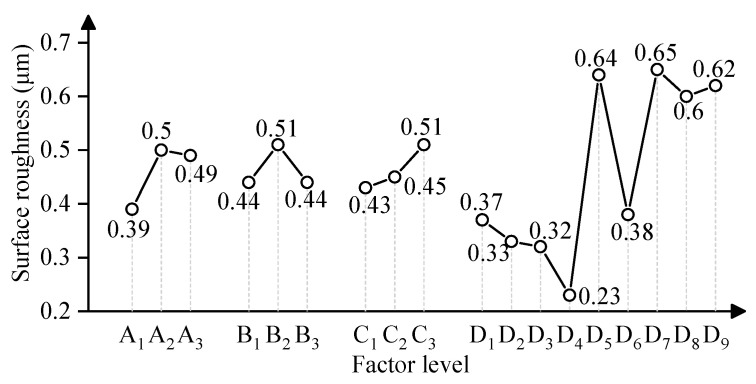
Effect of factor level on surface roughness.

Figure 6 shows the relationship between factor levels and surface roughness. The surface roughness of the 304V wire increases with the increase in abrasive volume. The high abrasive volume causes more abrasive to impact the wire, which results in an increase in surface roughness. The surface roughness increases first and then decreases with the increase in air pressure and lift-off height. As the mesh size of the abrasive decreases, the surface roughness decreases first and then remains stable. The small abrasive size has a low impact energy, which generates a small pit size. However, the small size causes more abrasive to impact the wire and causes the pits number increase in the surface, which keeps the surface roughness stable.

### 3.4. Wire Mechanical Properties After Abrasive Blasting

**Figure 7:**
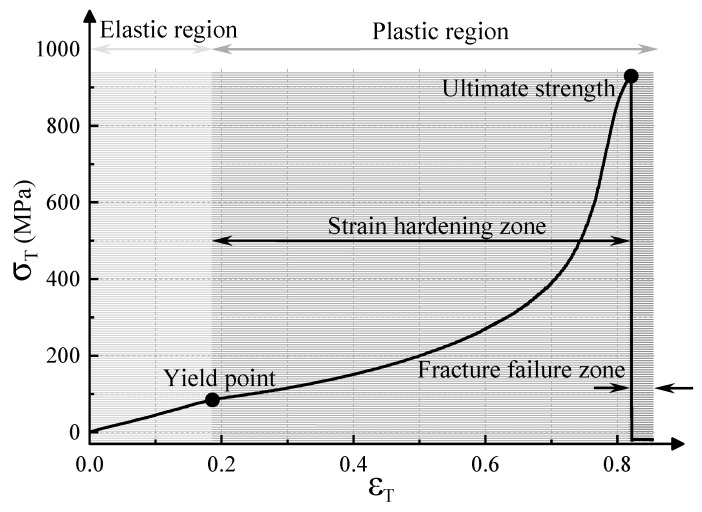
The stress-strain curve of the wire component.

Figure 7 presents the relationship between the wire component stress (σ_T_) and strain (ε_T_) in the tensile process, which goes through an elastic deformation stage and a plastic deformation stage with stress hardening and fracturing. The yield strength and tensile strength of the wire component are 87.4 MPa–96.2 MPa and 879.9 MPa–933.7 MPa before the abrasive blasting.

**Figure 8:**
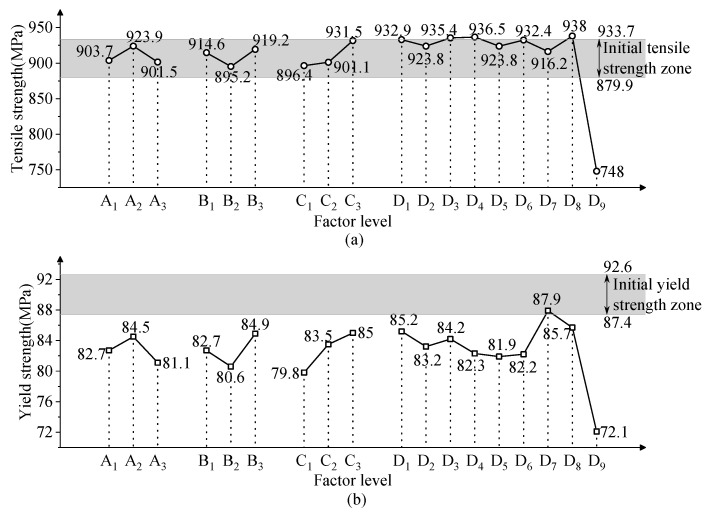
Effect of factor level on mechanical properties: (**a**) tensile and (**b**) yield strength.

The tensile strength and yield strength of the wire after sandblasting are given in Table 2. Figure 8a shows the relationship between the factor level and the wire tensile strength. The air pressure, lift-off height, and abrasive volume do not affect the tensile strength of the wire. The use of the alumina abrasive reduces the tensile strength by about 28% through the removal of the wire material. Other abrasives do not affect the wire tensile strength. The relationship between the wire yield strength and factor level is shown in Figure 8b. Obviously, the process parameters of abrasive blasting reduce the yield strength and weaken the ability of the wire to resist plastic deformation.

### 3.5. Range Analysis

According to Equations (1) and (2), the *K*, $\bar{K}$, and *R* of the processing time, surface roughness, tensile strength, and yield strength are calculated, as presented in Table 3. According to the order of the R, the primary and secondary factors affecting the processing time and surface roughness of the 304V wire are the abrasive type and air pressure, followed by the abrasive volume and lift-off height. The primary and secondary factors that affect the mechanical properties are the abrasive type and abrasive volume, followed by the lift-off height and air pressure.

### 3.6. Damage Quantity

**Figure 9:**
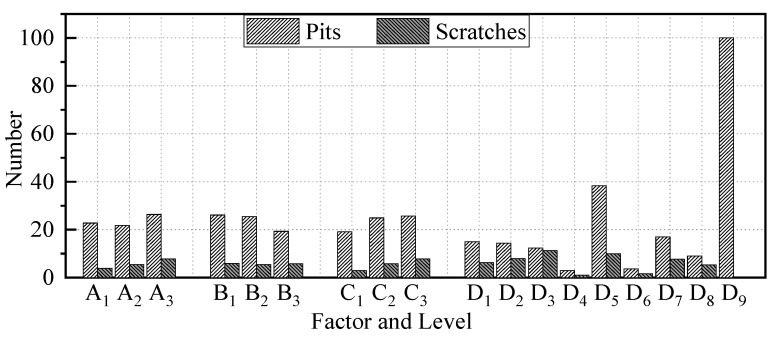
Surface damage quantity on stainless steel wire components.

From the surface observation, the main damage after blasting comprises pits and scratches. After sandblasting, ultrasonic cleaning is used to clean the wire assembly to ensure that there is no abrasive inside the pits and scratche. The number of pits and scratches on the 304V wire surface after abrasive blasting were counted, as shown in Figure 9. The quantity of pits is significantly higher than that of scratches under all conditions, which indicates that the impact pit is the primary removal mode in abrasive blasting.

With the increase in air pressure, the number of scratches increases due to the increased energy of the abrasive. The pits also show an increasing trend, even if the A2 has the lowest quantity. The lift-off height shows that the number of pits on the 304V surface is negative in proportion to the lift-off height and positive in proportion to the abrasive volume. The number of scratches is positively correlated to the air pressure, lift-off height, and abrasive volume and negatively correlated to the abrasive size. The larger mesh size can reduce pit quantity and increase scratch quantity due to the high velocity in the smaller mass, based on the resin abrasive results (D_1_, D_2_, and D_3_). For the plastic abrasive, the removal ability of the TPU (D_4_) and PTFE (D_6_) abrasive is poor, which left much fewer pits and scratches on the surface. The pits and scratches on the surface processed by the PVC (D_5_) abrasive are much larger than D_4_ and D_6_, which shows the high removal efficiency. The scratches for corn (D_7_) are larger than those for walnut (D_8_), which results in a high material removal rate. However, the roughness of the workpiece is larger. The alumina abrasive (D_9_) shows the most pits and least scratches on the wire surface among all the abrasive materials, which indicates the high material removal ability.

### 3.7. Material Removal Mechanism

**Figure 10:**
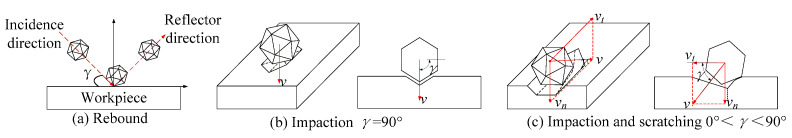
Material removal mechanism in abrasive blasting.

During the abrasive blasting process, the abrasive impacts the solder and the 304V components. Under different impact parameters and angles, different material removal mechanisms can be observed, as shown in Figure 10.

(1) When the hardness and Young’s modulus of the abrasive are much smaller than the workpiece, a rebound occurs after the abrasive impacts the surface.

(2) With the increase in the abrasive hardness and Young’s modulus, the impact causes plastic deformation of the workpiece. Under γ = 90°, impact pits are left on the surface. Under 0° < γ < 90°, the *v*_t_ mat induces scratch marks on the surface.

**Figure 11:**
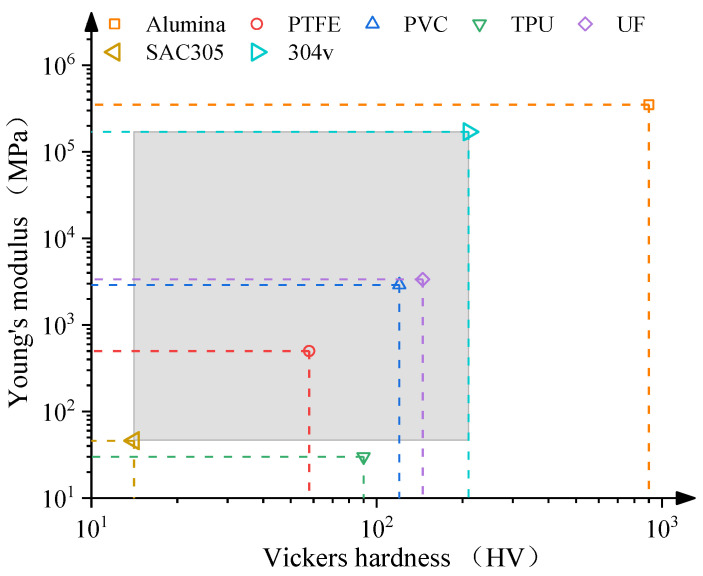
Young’s modulus and hardness of the experimental materials.

Figure 11 presents Young’s modulus and hardness comparison of the five abrasive types, SAC305, and the 304V wire. Due to the mechanical properties of the biomass material changing with the moisture and biostructure, which is hard to determine, the corn and walnut properties are excluded in Figure 11. Young’s modulus is a physical parameter that describes the ability to resist elastic deformation. Hardness is the ability to resist plastic deformation. Young’s modulus and hardness of SAC305 are 14.1 HV and 46 × 10^5^ MPa. Young’s modulus and hardness of 304V are 210 HV and 1.7 × 10^5^ MPa. To achieve the different cuttings of the SAC305 and 304V, the plastic deformation should occur in SAC305, and elastic deformation should occur in 304V. Based on the above analysis, the ranges for the different cuttings are marked by the light gray rectangle. Young’s modulus of TPU is lower than that in SAC305, which indicated that more elastic deformation occurred in the TPU after impact, which consumes the most energy and results in poor removal ability. The hardness of TPU is between the SAC305 and 304V, which shows the plastic deformation of the SAC305, which is validated in Figure 3b with many pits on the SAC305 surface. The alumina abrasive has a higher Young’s modulus and hardness than 304V, which directly results in the removal of the wire, as shown in Figure 4c.

The properties of UF resin, PVC, and PTFE are located in the gray rectangle, which means all of them can obtain a different cutting. The UF resin has the highest Young’s modulus and hardness, which shows the fastest removal rate. The properties of PVC are close to the UF resin, which shows that the solder can also be removed. Young’s modulus and hardness of PVC is lower than that of the UF resin, resulting in a lower efficiency, as shown in Figure 3b. The PTFE has a much lower Young’s modulus and hardness, which results in a much lower removal rate and longer processing time. Based on the results above, the high abrasive hardness and Young’s modulus decrease the processing time, resulting in a higher removal efficiency.

When Young’s modulus and the hardness of the abrasive are lower than that in SAC305, a high degree of elasticity occurs in the abrasive, which consumes the majority of the impact energy, causing the abrasive rebound. The effect on SAC is weak and cannot easily cause the removal. When Young’s modulus and hardness are in the gray rectangle, the impact of the abrasive damages the SAC directly without causing failure of the stainless steel wire. Under this range, the higher Young’s modulus and hardness can achieve a higher material removal rate and lower processing time. If Young’s modulus and the hardness of the abrasive are higher than 304V, the removal of 304V is inevitable, which causes the tensile strength to be reduced. Abrasive blasting causes defects such as pits and scratches on the wire, which change the wire’s integrity and reduce the yield strength. During blasting, the abrasive directly acts on the surface of the workpiece to achieve material removal. The differences in abrasive material properties, size, and shape lead to different material removal mechanisms, which results in different processing times and surface roughness. The air pressure determines the abrasive energy. The abrasive volume and the lift-off height affect the abrasive quantity per unit time impacted on the 304V wire. The effect of abrasive volume is much larger than the lift-off height, which results in a stronger processing time and surface roughness change.

## 4. Conclusions

In this study, experiments were conducted to reveal the abrasive blasting mechanism. The effects of air pressure, lift-off height, abrasive volume, and abrasive type on the processing time and surface roughness are systematically discussed. The conclusions are as follows:

(1) The different cutting can be achieved as the abrasive hardness and Young’s modulus are in the middle of the SAC305 solder and 304V stainless steel. The high hardness and Young’s modulus can improve the blasting efficiency. Under the six types of abrasive, the resin is the best for removing the SAC305 solder and maintaining the integrity of the 304V with high efficiency.
(2) The primary and secondary factors affecting the surface roughness and processing time are the abrasive type and air pressure, followed by the abrasive volume and lift-off height. The primary and secondary factors that affect the mechanical properties are the abrasive type and abrasive volume, followed by the lift-off height and air pressure.
(3) The processing time decreases with the increase in air pressure, abrasive hardness, and Young’s modulus. Abrasive blasting surface roughness increases with the increase in the abrasive volume. The tensile strength of the wire is affected by the abrasive Young’s modulus and hardness, and the yield strength decreases due to the blasting.
(4) When using the resin abrasive, the solder can be removed without damaging the stainless steel wire substrate by adjusting the process parameters.
(5) The impact pits are the primary material removal mode in abrasive blasting. The quantity of pits on the surface has a negative relationship with the lift-off height and abrasive mesh size. The quantity of scratches has a positive correlation to air pressure, lift-off height, and abrasive volume, and a negative correlation to the abrasive size.

## Figures and Tables

**Table 1:** Material parameters used in the experiment.

Name	Material	Size	Density (g·cm^−3^)	Young’s Modulus (MPa)	Vickers Hardness (HV)	Figure
Wire component	304V	Ø0.65 mm	7.93	1.7 × 10^5^	210	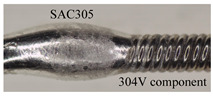
Solder	SAC305	0.6 mm^3^	7.37	46	14.1	
Resin	Urea formaldehyde resin	#120, #150, #200	1.2	3358	145–175	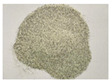
Plastic abrasive	TPU	#300	1.1	30	90–95	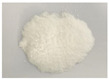
	PVC	#200	1.38	2900	120–125	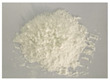
	PTFE	#300	2.1–2.3	500	58	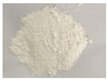
Biomass	Corn	#200	0.37	-	-	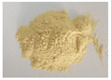
	Walnut	#200	0.24	-	-	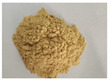
Industry	Alumina	#200	3.85	3.5 × 10^5^	900	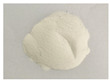

**Table 2:** Orthogonal experiment.

No.	Factor Level	Pressure (MPa)	Lift-Off Height (mm)	Abrasive Volume (mm)	Abrasive	Times (s)	Surface Roughness (μm)	Tensile Strength (MPa)	Yield Strength (MPa)
1	A_1_B_1_C_1_D_1_	0.3	2	60	#120 resin	160	0.19	926.1	77.5
2	A_1_B_1_C_1_D_2_	0.3	2	60	#150 resin	120	0.21	946.3	82.3
3	A_1_B_1_C_1_D_3_	0.3	2	60	#200 resin	150	0.25	896.7	83.9
4	A_1_B_2_C_3_D_4_	0.3	4	100	TPU	300	0.16	947.9	82.5
5	A_1_B_2_C_3_D_5_	0.3	4	100	PVC	300	1.02	917.4	79.4
6	A_1_B_2_C_3_D_6_	0.3	4	100	PTFE	300	0.16	931.5	84.8
7	A_1_B_3_C_2_D_7_	0.3	6	80	Corn	300	0.54	984.0	96.1
8	A_1_B_3_C_2_D_8_	0.3	6	80	Walnut	300	0.22	944.4	89.1
9	A_1_B_3_C_2_D_9_	0.3	6	80	Alumina	10	0.83	721.3	68.8
10	A_2_B_1_C_3_D_9_	0.4	2	100	Alumina	5	0.44	881.2	86.8
11	A_2_B_1_C_3_D_7_	0.4	2	100	Corn	180	0.77	940.7	81.5
12	A_2_B_1_C_3_D_8_	0.4	2	100	Walnut	180	0.86	929.8	89.1
13	A_2_B_2_C_2_D_3_	0.4	4	80	#200 resin	50	0.29	922.7	82.9
14	A_2_B_2_C_2_D_1_	0.4	4	80	#120 resin	170	0.62	933.3	90.4
15	A_2_B_2_C_2_D_2_	0.4	4	80	#150 resin	90	0.34	909.7	80.3
16	A_2_B_3_C_1_D_6_	0.4	6	60	PTFE	300	0.67	938.0	83.1
17	A_2_B_3_C_1_D_4_	0.4	6	60	TPU	300	0.37	941.4	90.7
18	A_2_B_3_C_1_D_5_	0.4	6	60	PVC	300	0.16	944.7	75.5
19	A_3_B_1_C_2_D_5_	0.5	2	80	PVC	300	0.74	909.2	90.9
20	A_3_B_1_C_2_D_6_	0.5	2	80	PTFE	300	0.33	927.7	78.8
21	A_3_B_1_C_2_D_4_	0.5	2	80	TPU	300	0.16	920.2	73.8
22	A_3_B_2_C_1_D_8_	0.5	4	60	Walnut	245	0.74	939.9	78.8
23	A_3_B_2_C_1_D_9_	0.5	4	60	Alumina	5	0.60	641.4	60.6
24	A_3_B_2_C_1_D_7_	0.5	4	60	Corn	190	0.64	929.5	86.1
25	A_3_B_3_C_3_D_2_	0.5	6	100	#150 resin	40	0.44	935.4	87.1
26	A_3_B_3_C_3_D_3_	0.5	6	100	#200 resin	35	0.42	986.9	85.7
27	A_3_B_3_C_3_D_1_	0.5	6	100	#120 resin	100	0.32	939.4	87.8

**Table 3:** The range of experimental results by factor level.

Factor Level	Times (s)			Surface Roughness (μm)			Tensile Strength (MPa)			Yield Strength (MPa)		
	*K*	$\bar{K}$	*R*	*K*	$\bar{K}$	*R*	*K*	$\bar{K}$	*R*	*K*	$\bar{K}$	*R*
A1	1940	215.56	47.22	3.55	0.39	0.11	8132.9	903.7	22.4	744.4	82.7	3.4
A2	1575	175.00		4.53	0.50		8314.9	923.9		760.3	84.5	
A3	1515	168.33		4.39	0.49		8113.3	901.5		729.6	81.1	
B1	1695	188.33	5.00	3.95	0.44	0.07	8231.3	914.6	24.0	744.6	82.7	4.3
B2	1650	183.33		4.56	0.51		8057.0	895.2		725.8	80.6	
B3	1685	187.22		3.97	0.44		8272.8	919.2		763.9	84.9	
C1	1770	196.67	42.22	3.83	0.43	0.08	8067.7	896.4	35.1	718.5	79.8	5.2
C2	1820	202.22		4.06	0.45		8109.8	901.1		751.1	83.5	
C3	1440	160.00		4.59	0.51		8383.6	931.5		764.7	85.0	
D1	430	143.33	293.33	1.12	0.37	0.42	2798.8	932.9	190.0	255.7	85.2	15.8
D2	245	81.67		0.99	0.33		2771.4	923.8		249.7	83.2	
D3	240	80.00		0.96	0.32		2806.3	935.4		252.5	84.2	
D4	900	300.00		0.68	0.23		2809.5	936.5		247	82.3	
D5	900	300.00		1.92	0.64		2771.3	923.8		245.8	81.9	
D6	900	300.00		1.15	0.38		2797.2	932.4		246.7	82.2	
D7	670	223.33		1.96	0.65		2748.6	916.2		263.7	87.9	
D8	725	241.67		1.81	0.60		2814.1	938.0		257.0	85.7	
D9	20	6.67		1.87	0.62		2243.9	748.0		216.2	72.1	

## Data Availability

The original contributions presented in the study are included in the article, further inquiries can be directed to the corresponding authors.
